# *SWG5* regulates grain size and weight via sugar metabolism-mediated signaling in rice

**DOI:** 10.3389/fpls.2025.1552268

**Published:** 2025-03-24

**Authors:** Wenhui Lu, Gaoyi Cai, Yannan Xing, Xingzhe Fu, Lingling Zhou, Yijun Tang, Ran Xu, Yunhai Li, Lian Wu

**Affiliations:** ^1^ School of Breeding and Multiplication (Sanya Institute of Breeding and Multiplication), Hainan University, Sanya, China; ^2^ Zunyi Normal College, Department of Resources and Environment, Zunyi, Guizhou, China; ^3^ State Key Laboratory of Plant Cell and Chromosome Engineering, Chinese Academy of Sciences (CAS) Centre for Excellence in Molecular Plant Biology, Institute of Genetics and Developmental Biology, Chinese Academy of Sciences, Beijing, China

**Keywords:** rice, SWG5, grain size, sugar signaling, grain weight

## Abstract

Grain size significantly affects rice yield and quality. Although several genes that regulate grain size have been identified, their mechanisms remain unclear. In this study, we characterized the *swg5* mutant, which has a smaller plant height, shorter panicles, and smaller grains compared to the wild type (WT). MutMap resequencing and gene knockout analysis identified *SWG5*, a gene encoding the kinesin-13a protein, a new allele of *SRS3* that positively regulates grain length and weight. RNA sequencing analyses revealed that the *SWG5* allele is involved in diterpenoid biosynthesis, amino sugar metabolism, and pentose-glucuronate interconversions. Furthermore, young panicles of the *swg5* mutant exhibited decreased sucrose invertase activity as well as reduced sugar and starch content. These findings indicate that *SWG5/SRS3* plays a significant role in sugar metabolism, influencing grain size and weight in rice. This research provides valuable insights into breeding rice varieties with improved yield and grain quality.

## 1 Introduction

Rice (*Oryza sativa L.*) is the most important food crop globally, feeding over half of the worldwide population (Zuo and Li, 2014). Enhancing rice yield is crucial for food security, economic development, and environmental sustainability (Hashim et al., 2024). Rice yield is determined by four main factors: effective panicle number, grain number per panicle, grain filling rate, and grain weight, with the latter being associated with grain size. Grain size significantly influences rice yield and appearance quality (Ren et al., 2023; Zhao et al., 2022). Thus, identifying genes that regulate grain size and understanding their mechanisms is essential for breeding high-yield, high-quality rice varieties.

In recent years, significant progress has been made in understanding the molecular mechanisms governing rice grain size. Researchers have identified key genes that influence the development of rice grain size, shape, and weight. These genes impact grain development through various pathways, including hormone metabolism, G protein signaling, mitogen-activated protein kinase (MAPK) signaling, and the ubiquitin–proteasome pathway (Li et al., 2018; Li et al., 2019). However, the molecular regulatory mechanisms of rice grain size are not yet fully understood.

Sugars are the main products of photosynthesis and are essential for rice growth and development. Mutations in the *PHS8* gene lead to the accumulation of small sugars in the endosperm. This accumulation inhibits the expression of *OsABI3* and *OsABI5* in the ABA signaling pathway, affecting seed germination (Du et al., 2018). Knocking out the *OsSTP15* gene reduces glucose efflux and enhances sugar transport from the source leaves to the stem base. As a result, levels of glucose, sucrose, and trehalose-6-phosphate increase at the stem base. This rise in sugar levels boosts cytokinin (CK) synthesis and signaling, promoting tillering and increasing rice yield (Li et al., 2024). Overexpressing the *OsNAC23* gene raises 6-phospho-trehalose levels in source organs, improving photosynthesis and supporting the development of storage organs. This process leads to a high rice yield (Li et al., 2022). Recent studies have identified genes that regulate rice grain size through sugar signaling. For example, *OsFRK3* decreases maltose levels while increasing sucrose and fructose levels, which improves grain size and weight (Zhang Z. et al., 2023). The *GF14f* gene negatively regulates grain size (Song et al., 2024; Zhang et al., 2019). Additionally, *FGW1* interacts with *GF14F* to regulate grain width by affecting cell processes and sugar metabolism (Li et al., 2023). The *SMS2* gene encodes vacuolar acid invertase. Mutations in this gene reduce the sugar content in rice grains, resulting in smaller grains (Huang et al., 2024).

Kinesin-13 proteins are important for plant development, stress responses, and the regulation of grain size. The *SRS3* gene, which belongs to the kinesin-13 subfamily, encodes a protein made up of 819 amino acids. This protein includes a motor domain and a coiled-coil structure. *SRS3* affects grain size by influencing the cell length in the spikelet hull (Kitagawa et al., 2010). An allelic variant of *SRS3*, *SGL*, regulates the height of rice plants through gibberellin (GA) signaling (Wu et al., 2014). Recent studies have shown that *SRS3/BSH1* acts downstream of *BRI1* (BRASSINOSTEROID INSENSITIVE 1) to negatively regulate brassinosteroid (BR) signaling and plant architecture in rice (Zhang et al., 2022). *SRS3* also forms a complex with OsTUB1, which stabilizes the Na^+^ transporter OsHKT1;5. This stabilization helps maintain ion balance and enables rice plants to cope with salt stress (Chen et al., 2022). These studies demonstrated that kinesin-13 proteins significantly influence rice growth, development, and stress responses. However, the exact mechanisms by which they affect agronomic traits remain unclear.

In this study, we identified and characterized the rice mutant small and wide grain 5 (*swg5*), which exhibits a short grain length and increased grain width. Using MutMap technology, we cloned the *SWG5* gene and found that it encodes the kinesin-13a protein, allelic to the *SRS3* gene. Although *SRS3* is known to regulate grain size, its molecular mechanism remains poorly understood. We further investigated the molecular mechanism of *SWG5* in regulating grain size and weight in rice. Our results demonstrated that *SWG5* positively regulates grain length and weight while negatively regulating grain width by influencing sucrose metabolism. This study enhances our understanding of the regulatory role of *SWG5/SRS3* in determining grain size.

## 2 Materials and methods

### 2.1 Plant material and growth conditions

The wild-type material utilized in this study was the japonica rice variety ZH11 (WT). The *swg5* mutant, characterized by small and wide grains, was derived from ZH11 treated with EMS. CRISPR-Cas9 technology was employed to generate the *swg5-cr1* and *swg5-cr2* mutants. All rice materials were cultivated in the natural field environments of Lingshui (N18°, E110°) and Sanya (N18°, E108°), Hainan Province, China, from 2022 to 2024.

### 2.2 Agronomic trait measurement

During the maturity stage, 10 plants each from the WT and *swg5* groups were selected to measure plant height and tiller number. Photographs of the plants were taken using a digital camera. After harvesting, the plants were stored indoors for three months. For analysis, 10 mature panicles from each group were assessed for panicle length, grain number, seed setting rate, and the number of primary and secondary branches. Additionally, mature seeds were measured for grain length, width, and weight using a ScanMaker i800 (MICROTEK, China). Photographs of the panicles and seeds were also taken.

### 2.3 Histological analysis

A scanning electron microscope (S-3000N & Quorum PP3000T, Hitachi, Japan) was employed to capture images of the outer surface of the mature seed glume. ImageJ software (Schneider et al., 2012) was subsequently utilized to quantify and measure the cells on the glume surface. The protocols for observing and measuring cell number and size were consistent with those described in our previous study (Duan et al., 2015).

### 2.4 Identification of the *SWG5* gene

We crossed ZH11 with the *swg5* mutant at the heading stage to generate an F_2_ population and cloned the *SWG5* gene. From this F_2_ population, we selected 50 rice plants exhibiting the *swg5* phenotype, pooled their DNA in equal amounts, and conducted whole-genome resequencing using NextSeq 550 (Illumina). We then used the MutMap method to isolate the *SWG5* gene (Abe et al., 2012) and analyzed the SNP-to-INDEL ratio, consistent with our previous studies (Fang et al., 2016). The *swg5* mutation was identified by PCR amplification of the *SWG5* genomic region in both wild-type and *swg5* mutant plants, followed by sequencing. cDNA was amplified by PCR and analyzed by electrophoresis to assess the effect of *SWG5* on alternative splicing. The primer sequences are listed in **Supplementary Table S1**.

### 2.5 Plasmid construction and genetic transformation

We employed CRISPR/Cas9 gene editing to modify the *SWG5* gene in the japonica variety ZH11 wild type (WT) and elucidate the role of *SWG5* in rice grain development. This procedure resulted in the creation of *SWG5* knockout mutants *swg5-1* (1-bp insertion) and *swg5-2* (2-bp deletion). The design of the target gene, vector construction, and transformation methods were performed according to previously established protocols (Zhang et al., 2022).

### 2.6 RNA-seq analysis

Total RNA was extracted from 5-cm young panicles of ZH11 (WT) and *swg5-cr1* using the RNA Prep Pure Plant Kit (Tiangen, Beijing). Each sample comprised 20 plants, with three biological replicates prepared for library construction. Sequencing was conducted at Guangzhou Kidio Biotechnology Co., Ltd., using the Illumina NovaSeq 4000 platform. Quality control was performed using Fastp (v0.12.4) to filter low-quality data and obtain clean readings. The filtered reads were quantified and aligned to the Nipponbare Reference IRGSP-1.0 from the Ensembl Plants database using HISAT2 (v2.2.1) (Chen et al., 2018; Kim et al., 2019). Gene expression levels were quantified using FPKM, which estimates the number of fragments per kilobase of transcript per million mapped reads. Differential expression analysis was conducted using DESeq2 software, with edgeR used for comparisons between samples (Liao et al., 2019; Love et al., 2014). Genes or transcripts with a false discovery rate (FDR) below 0.05, |log2(fold-change)| > 0.5, and an adjusted p-value < 0.05 were considered significant.

GO and KEGG enrichment analyses were performed on differentially expressed genes in the young panicles of ZH11 and *swg5-cr1* using the clusterProfiler package in R (v4.3.2) to elucidate the mechanism of *SWG5* in regulating grain size. These analyses followed the methods described in our previous study (Huang et al., 2024).

### 2.7 RT-qPCR

We randomly selected seven differentially expressed genes to validate the RNA-seq results and investigate the role of *SWG5* in regulating rice grain size and weight through sugar signaling. We specifically chose three genes involved in sugar metabolism or synthesis (*Os05g0247100/OsHI-XIP*, *Os07g0539300*, and *Os06g0356800*) for confirmation by qRT-PCR. Specific primers were designed using the NCBI database, and the experiments were conducted in triplicate. The relative expression levels of the genes were calculated using the 2^−ΔΔCT^ method. The primer sequences are listed in **Supplementary Table S1**.

### 2.8 Analysis of invertase activity and sugar and starch contents

At the heading stage, we collected samples from young panicles (5-10 cm) of ZH11, *swg5-cr1*, and *swg5-cr2* to evaluate sugar and starch contents, as well as enzyme activity. Invertase activity [VIN (vacuolar invertase), CWIN (soluble cell wall invertase), CIN (cell wall invertase)] was measured using previously established methods (Morey et al., 2018). The sucrose and starch content in the young panicles (5–10 cm) was determined according to the protocols described in earlier studies (Li et al., 2013; Shu et al., 2014).

### 2.9 Statistical analysis

Data collation was performed using Microsoft Excel 2021, and statistical analyses were conducted using GraphPad Prism (v9.3.1.471). Two-tailed Student’s t-tests were utilized for comparisons between the two groups. One-way ANOVA was employed for comparisons among multiple groups. Graphs were generated using Adobe Photoshop CC 2020 and GraphPad Prism (v9.3.1.471).

## 3 Results

### 3.1 Phenotypic characterization of the *swg5* mutant

**Figure 1:**
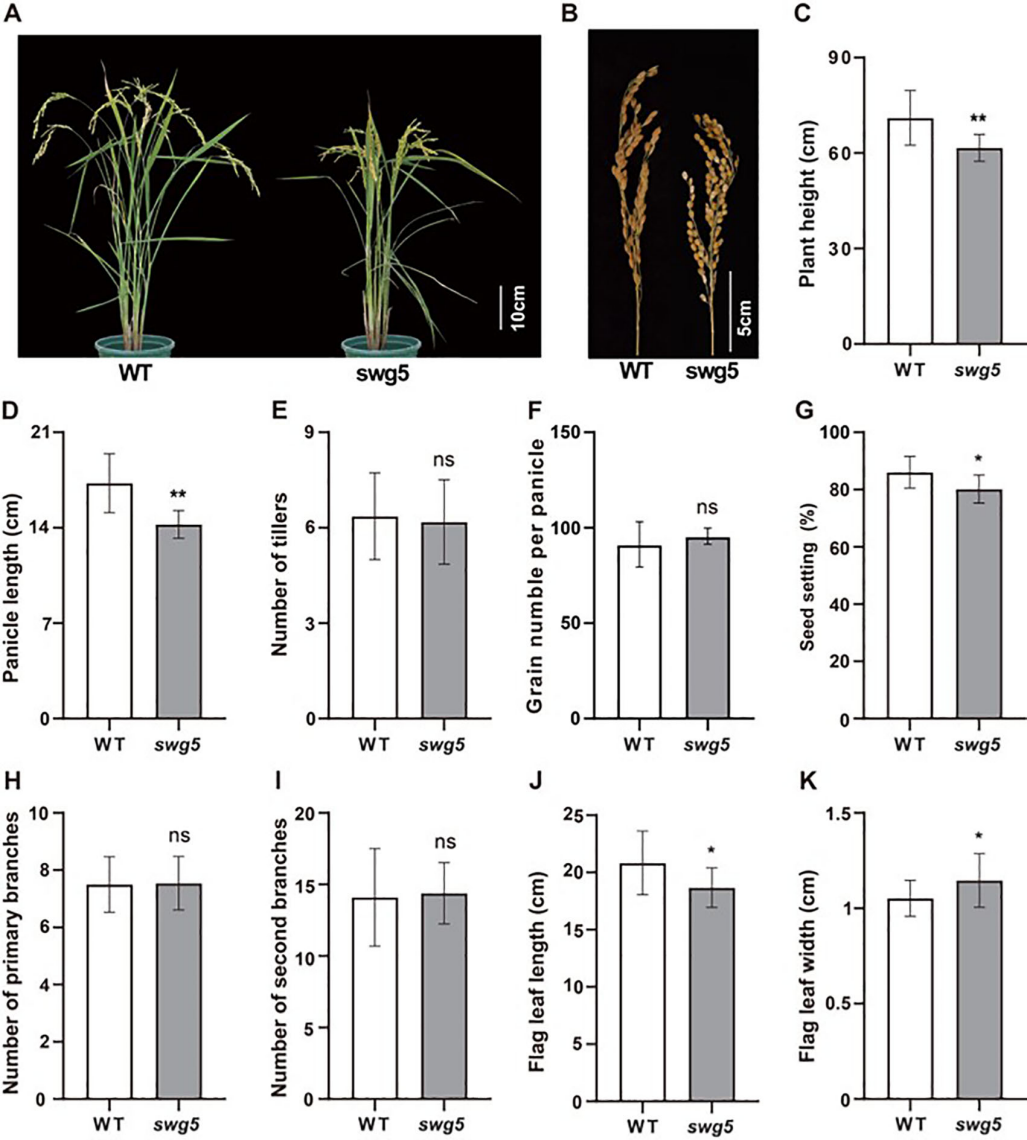
Phenotypic comparison of wild-type (WT) and *swg5* rice plants. **(A)** Whole plants; bar = 10 cm. **(B)** Panicles; bar = 5 cm. **(C)** Plant height. **(D)** Panicle length. **(E)** Panicle number. **(F)** Grain number per panicle. **(G)** Setting rate. **(H)** Primary branch number. **(I)** Secondary branch number. **(J)** Flag leaf length. **(K)** Flag leaf width. Values are means ± SD. Student’s *t*-test was used to calculate the p-values: *P < 0.05, **P < 0.01. NS indicates no significant difference.

We identified and characterized the rice mutant *swg5*, which exhibits reduced grain length and increased grain width, to better understand the genetic networks regulating grain size. At maturity, the *swg5* mutant displayed a significantly shorter plant height compared to WT (**Figures 1A, C**). The panicle length (**Figures 1B, D**), grain number per panicle (**Figure 1F**), and seed setting rate (**Figure 1G**) were all significantly reduced in the *swg5* mutant. At the same time, no significant changes were observed in the number of tillers (**Figure 1E**) or the primary and secondary branches (**Figures 1H, I**). Regarding leaf morphology, the flag leaf length of the *swg5* mutant was significantly shorter than that of the WT, while the flag leaf width was significantly greater (**Figures 1J, K**).

**Figure 2:**
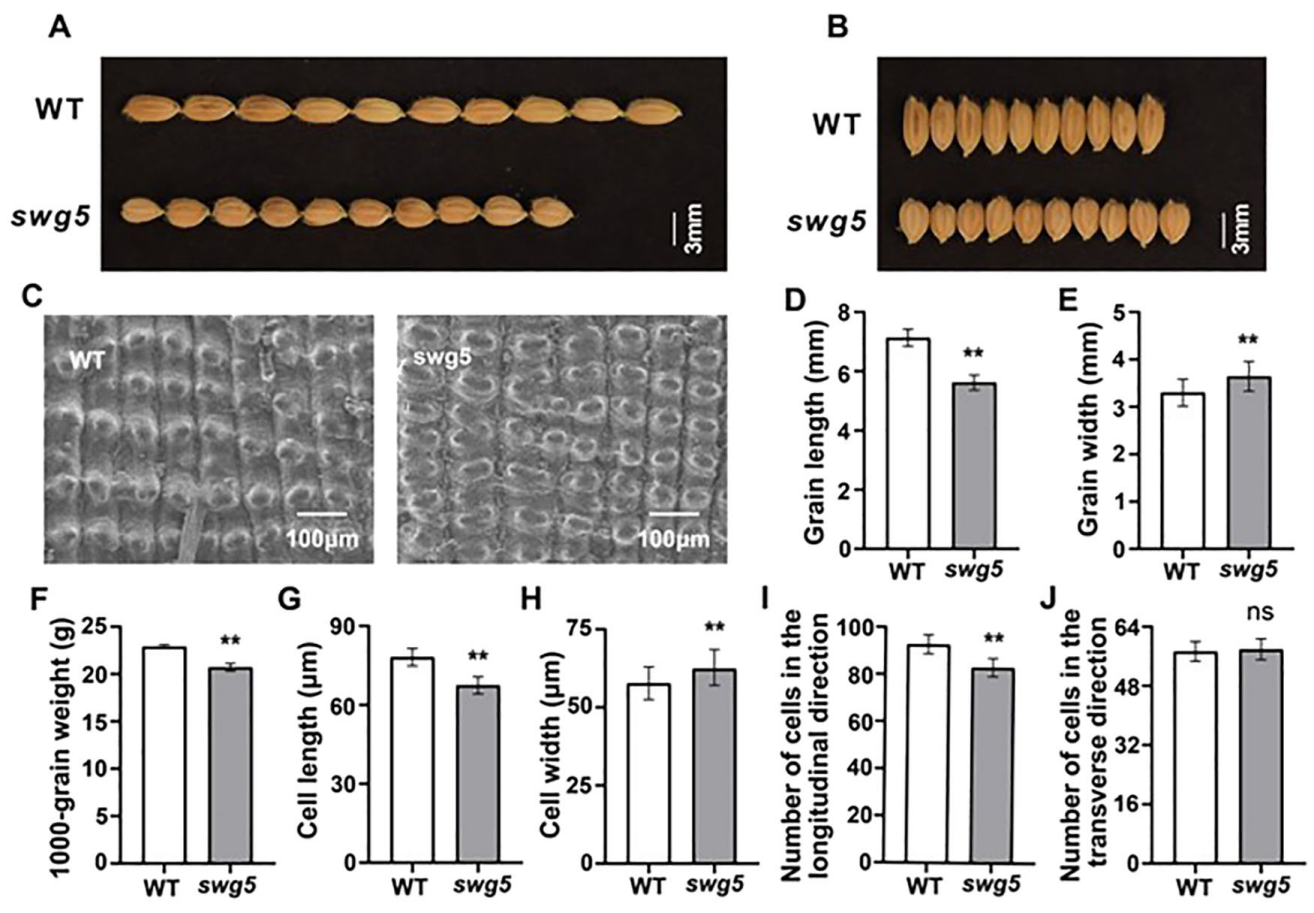
Morphological and histological analysis of spikelet hulls in WT and *swg5* to evaluate grain size. **(A-B)** Grain shape morphology in WT and *swg5*; bar = 3 mm. **(C)** Scanning electron microscopy of the glume outer surfaces of mature seeds; scale bars = 100 µm. **(D–F)** Grain length **(D)** grain width **(E)** and 1000 grain weight **(F)** in WT and *swg5*. **(G, H)** Average length **(G)** and width **(H)** of the outer epidermal cells of lemmas. **(I, J)** Number of outer epidermal cells in the longitudinal **(I)** and transverse **(J)** planes. Values are means ± SD. Student’s t-test was used to calculate the p-values; **P < 0.01. NS indicates no significant difference.

More importantly, we measured the mature seed traits of both the WT and *swg5* mutants. The grain length (**Figures 2A, D**) and 1000 grain weight (**Figure 2F**) of the *swg5* mutant were reduced by 22.54% and 9.56%, respectively, while the grain width (**Figures 2B, E**) increased by 9.35% compared to the WT. These results suggest that *SWG5* has a pleiotropic effect on both grain size and plant architecture.

### 3.2 *SWG5* regulates grain size by altering cell proliferation and cell expansion

We analyzed cell size and number in the spikelet hulls using scanning electron microscopy (SEM) to investigate the cellular basis for the short- and wide-grain traits in the *swg5* mutant (**Figure 2C**). The SEM images of the outer epidermis of mature grains showed that cell length was shorter and cell width was wider in the *swg5* mutant compared to WT (**Figures 2G, H**). Additionally, the number of cells with the same longitudinal length was significantly lower in the *swg5* mutant (**Figure 2I**). However, there was no significant difference in the number of cells with the same transverse length (**Figure 2J**). Specifically, the number of outer epidermal cells in the longitudinal planes was reduced by 11.1% in the *swg5* mutant. At the same time, the number of cells in the transverse planes showed no significant difference. These results suggest that the reduced grain length in the *swg5* mutant is due to few longitudinal cells, while the increased grain width was caused by large cell size.

### 3.3 *SWG5* is a novel mutation in the *SRS3* gene

**Figure 3:**
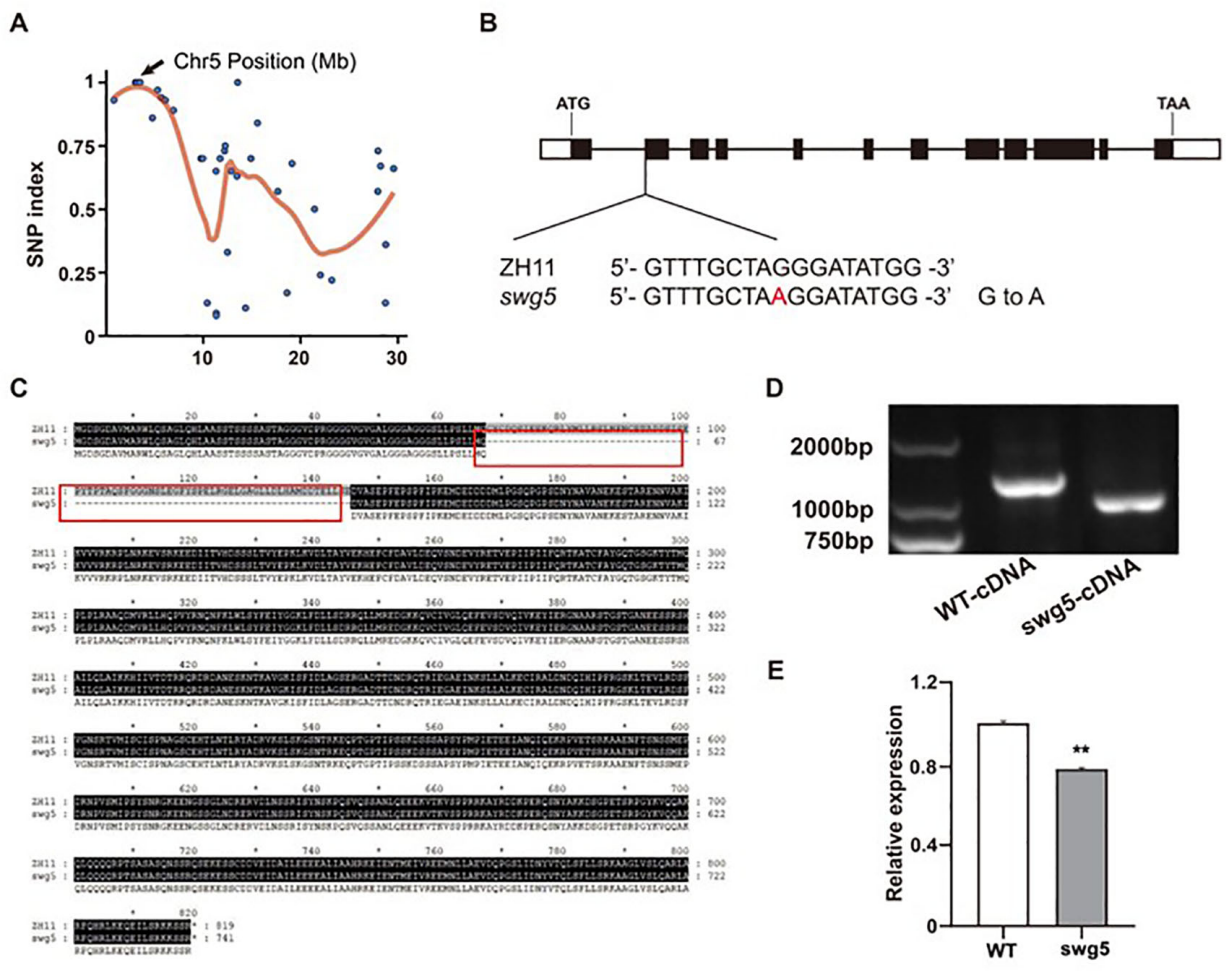
Identification of the *swg5* mutation. **(A)** Cloning of the *SWG5* gene using MutMap. **(B)** Structure of the *SWG5* gene showing the mutation site. **(C)** Comparison of amino acids between WT and the *swg5* mutant. **(D)** PCR analysis of the 234-bp splicing deletion in cDNA from three biological replicates. **(E)** Expression level of *SWG5* in WT and *swg5*. Values are means ± SD. Student’s t-test was used to calculate the p-values; **P < 0.01.

We crossed *swg5* mutants with the wild-type variety ZH11 to produce F_2_ populations to isolate *SWG5*. The phenotype of F_1_ heterozygous plants resembled that of WT. Analysis of the F_2_ population revealed a near 3:1 segregation ratio (normal: short and wide grains) for grain size phenotypes, suggesting that *swg5* is controlled by a single recessive gene. We isolated DNA from 50 F_2_ plants exhibiting the *swg5* mutant phenotype for whole-genome sequencing, with the ZH11 genome resequenced as a control. Potential causal mutations were identified following the method described by Huang et al. (2017). Six SNPs were linked to the small and wide grain phenotype of the *swg5* mutant. Of these, three occurred in coding regions but caused synonymous mutations. Notably, an SNP mutation was identified in the splice site region of *LOC_Os05g06280*, where a single base substitution from G to A was detected at site 787 in the intron, relative to the start codon (**Figures 3A, B**). This mutation in the splice site region primarily affects the splicing of precursor mRNA (pre-mRNA). Importantly, *LOC_Os05g06280/SRS3*, which encodes the kinesin-13a protein, has previously been reported to regulate rice grain length (Kitagawa et al., 2010; Wu et al., 2014). Therefore, we further sequenced and analyzed this mutation in *LOC_Os05g06280*. Sequence analysis revealed that the mutation, located near the start of exon 2, caused a 234-bp alternative splicing deletion in the coding sequence (**Figures 3C, D**). Additionally, gene expression analysis of WT and *swg5* mutants indicated that *LOC_Os05g06280* expression was significantly reduced in the mutant (**Figure 3E**). *LOC_Os05g06280/SRS3* was considered the putative candidate gene for *SWG5* based on these findings.

### 3.4 *SWG5* positively regulates grain length and weight

**Figure 4:**
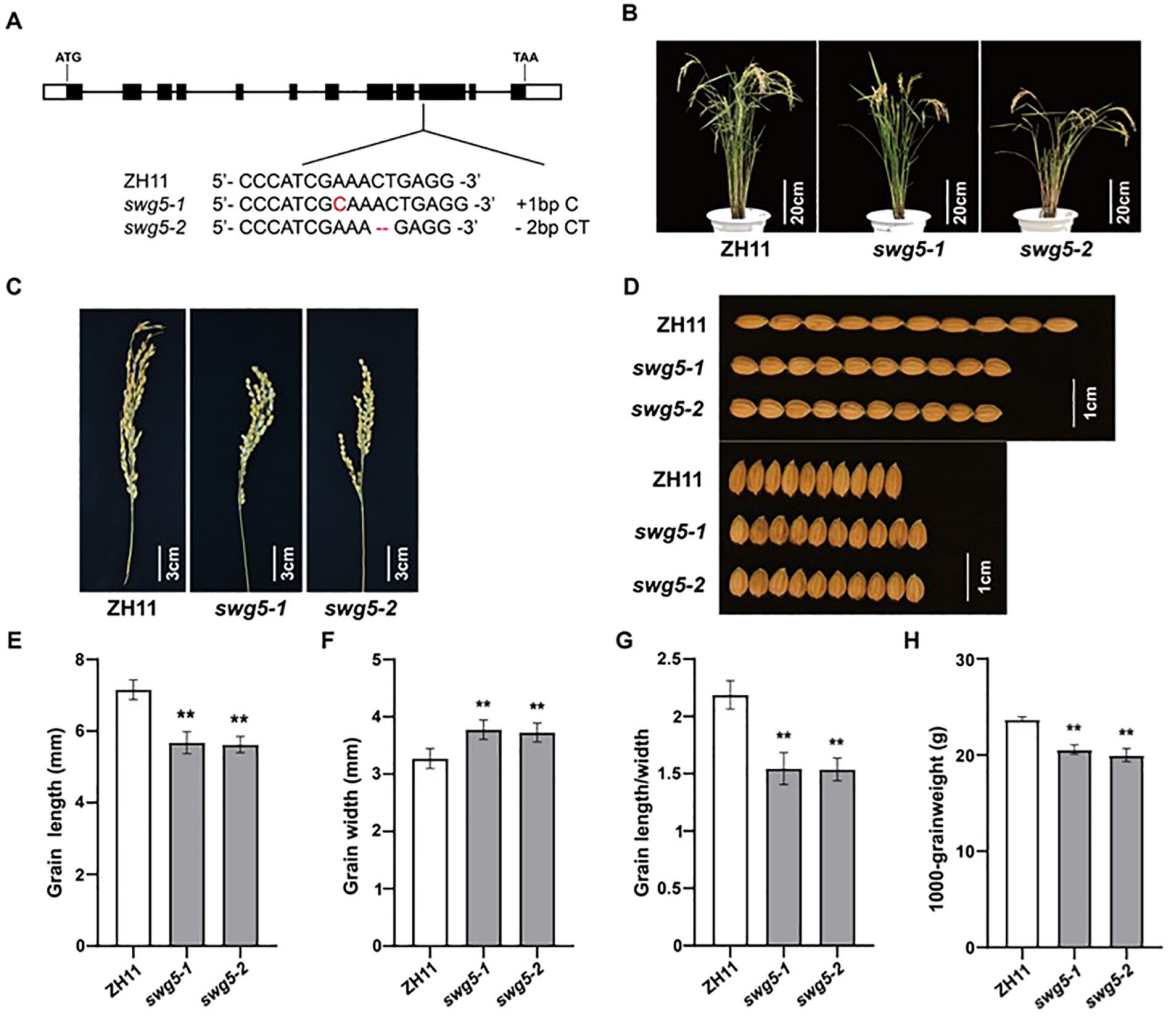
Gene knockouts of *SWG5*. **(A)** Mutant sequences of two homozygous mutants at the T_2_ generation. **(B, C)** Mature plants **(B)** and panicles **(C)** of ZH11, *swg5-1*, and *swg5-2*. Bars = 20 cm in **(B)** and 3 cm in **(C)**. **(D)** Grain length and width phenotypes of ZH11, *swg5-1*, and *swg5-2*. Bars = 1 cm **(E–H)** Grain length **(E)** grain width **(F)** grain length/width ratio **(G)** and 1000-grain weight **(H)** in ZH11, *swg5-1*, and *swg5-2*. Values are means ± SD. Student’s t-test was used for comparisons (n = 10 spikelets; **P < 0.01).

We used the CRISPR/Cas9 system to knock *SWG5* out in the ZH11 background to confirm that it was important for rice grain size. We created several transgenic plants with different mutations and selected two lines for analysis (**Figures 4A–C**): one with a 1-bp insertion (*swg5-1*) and the other with a 2-bp deletion (*swg5-2*). Grain size analysis showed that both mutants had a 20.63% and 20.6% reduction in grain length, while their grain widths increased by 13.31% and 12.16%, compared to ZH11 (**Figure 4D–F**). As a result, the grain shape index (grain length/width) decreased by 29.56% and 29.6% in the *swg5-1* and *swg5-2* mutants, respectively (**Figure 4G**). Thousand grain weight was reduced by 12.6% and 16.9%, respectively (**Figure 4H**). These results suggest that *SWG5* plays a key role in grain development, positively regulating grain length and weight while negatively regulating grain width.

### 3.5 GO and KEGG analyses of grain size regulation via *SWG5*

We conducted RNA sequencing (RNA-seq) on young panicles of ZH11 and *swg5-1* mutants to understand how *SWG5* regulates grain size. Among 231 differentially expressed genes (DEGs), 38.52% (89) were upregulated, and 61.47% (142) were downregulated in the *SWG5* knockout mutants compared to wild-type ZH11. We performed a functional analysis of these DEGs using GO and KEGG enrichment. The GO analysis identified 30 significantly enriched terms: 19 in biological processes, two in cellular components, and nine in molecular functions.

**Figure 5:**
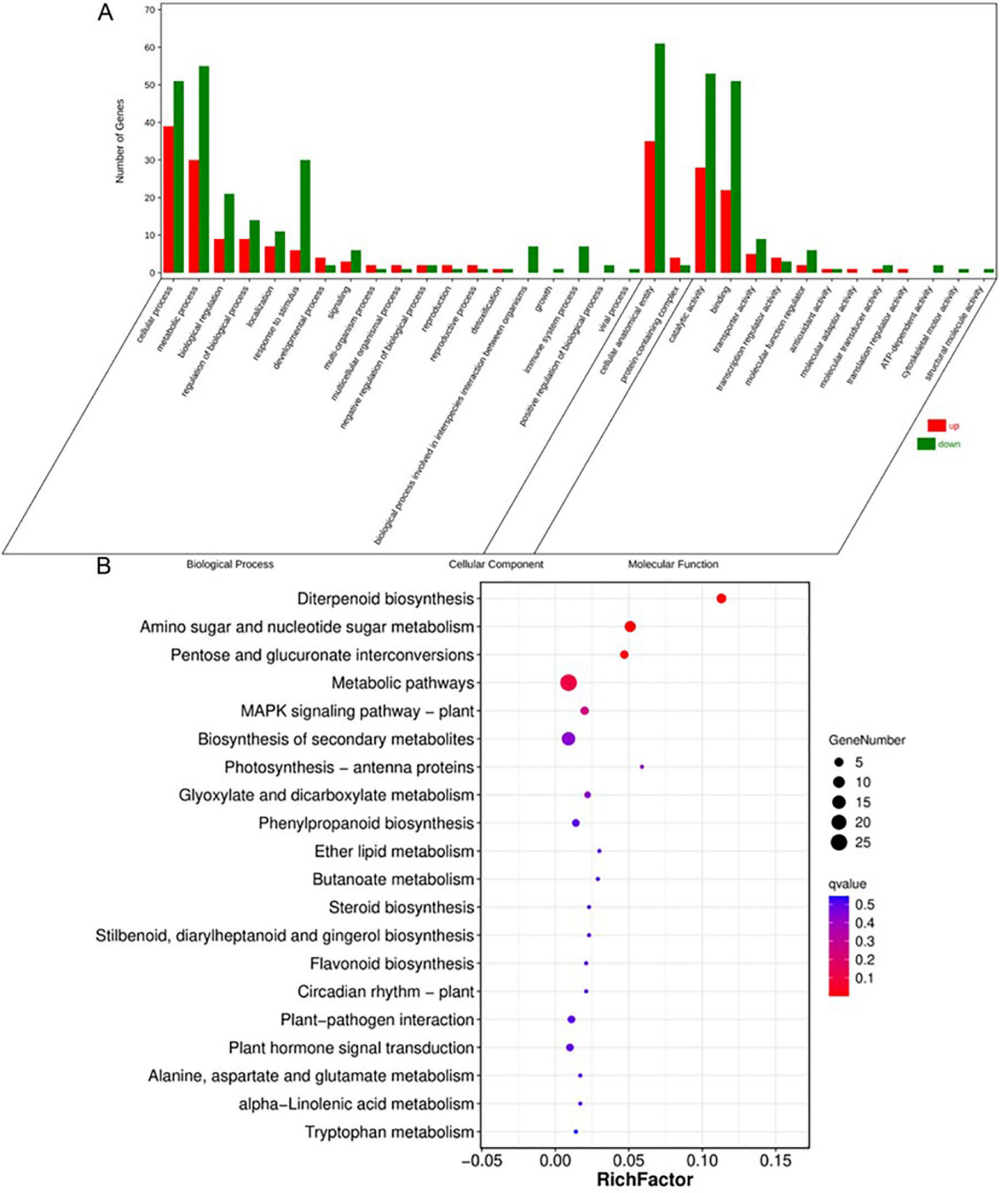
RNA-Seq analysis of grain size regulation by the *SWG5* gene. **(A)** Gene ontology enrichment analysis of differentially expressed genes (DEGs). **(B)** KEGG pathway analysis of differentially expressed genes (DEGs).

The top five biological process terms were cellular process, metabolic process, biological regulation, localization, and response to stimulus. In the cellular component category, the top two terms were cellular anatomical entity and protein-containing complex. The five high-ranking molecular function terms were catalytic activity, binding, transporter activity, transcription regulator activity, and molecular function regulator activity (**Figure 5A**). KEGG enrichment revealed that the five high-ranking metabolic pathways were diterpenoid biosynthesis, amino sugar and nucleotide sugar metabolisms, pentose and glucuronate interconversions, metabolic pathways, and the MAPK signaling pathway (**Figure 5B**). The remaining pathways had fewer DEGs than the previous ones, suggesting that *SWG5* regulates grain size through these five key pathways. Notably, amino sugar and nucleotide sugar metabolism, along with pentose and glucuronate interconversions, are involved in sugar metabolism. These results suggest that *SWG5* may regulate grain size by modulating sugar metabolism.

**Figure 6:**
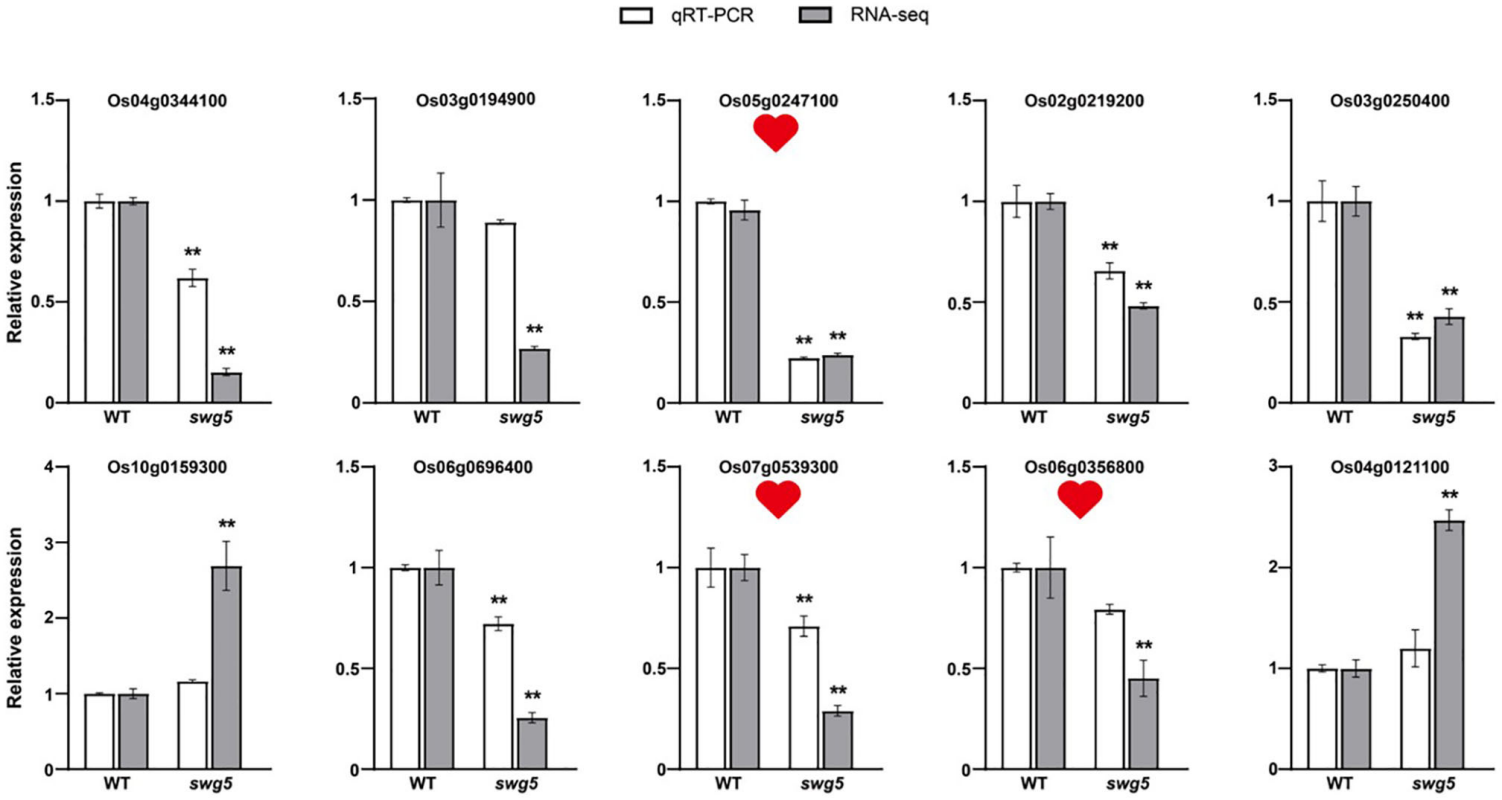
The differentially expressed genes between WT and *SWG5* were analyzed using qRT-PCR. The white and gray bars represent transcriptome data and quantitative data, respectively. The three red hearts indicate the genes involved in sugar metabolism. Values are means ± SD. Student’s t-test was used to calculate the p-values; **P < 0.01.

We randomly selected 10 genes (both upregulated and downregulated) from these pathways for qRT-PCR to validate the RNA-seq results. Three genes involved in sugar metabolism, transport, and synthesis were chosen: Os05g0247100/OsHI-XIP (xylanase inhibitory protein), Os07g0539300 (glucan Endo-1,3-beta-glucosidase precursor), and Os06g0356800 (glycosyl hydrolase). Specific primers were designed using Primer Premier (version 5.0). The qRT-PCR results confirmed that the gene expression patterns were consistent with the RNA-seq data, validating the transcriptome analysis (**Figure 6**).

### 3.6 *SWG5* positively regulates invertase activity and sugar accumulation

**Figure 7:**
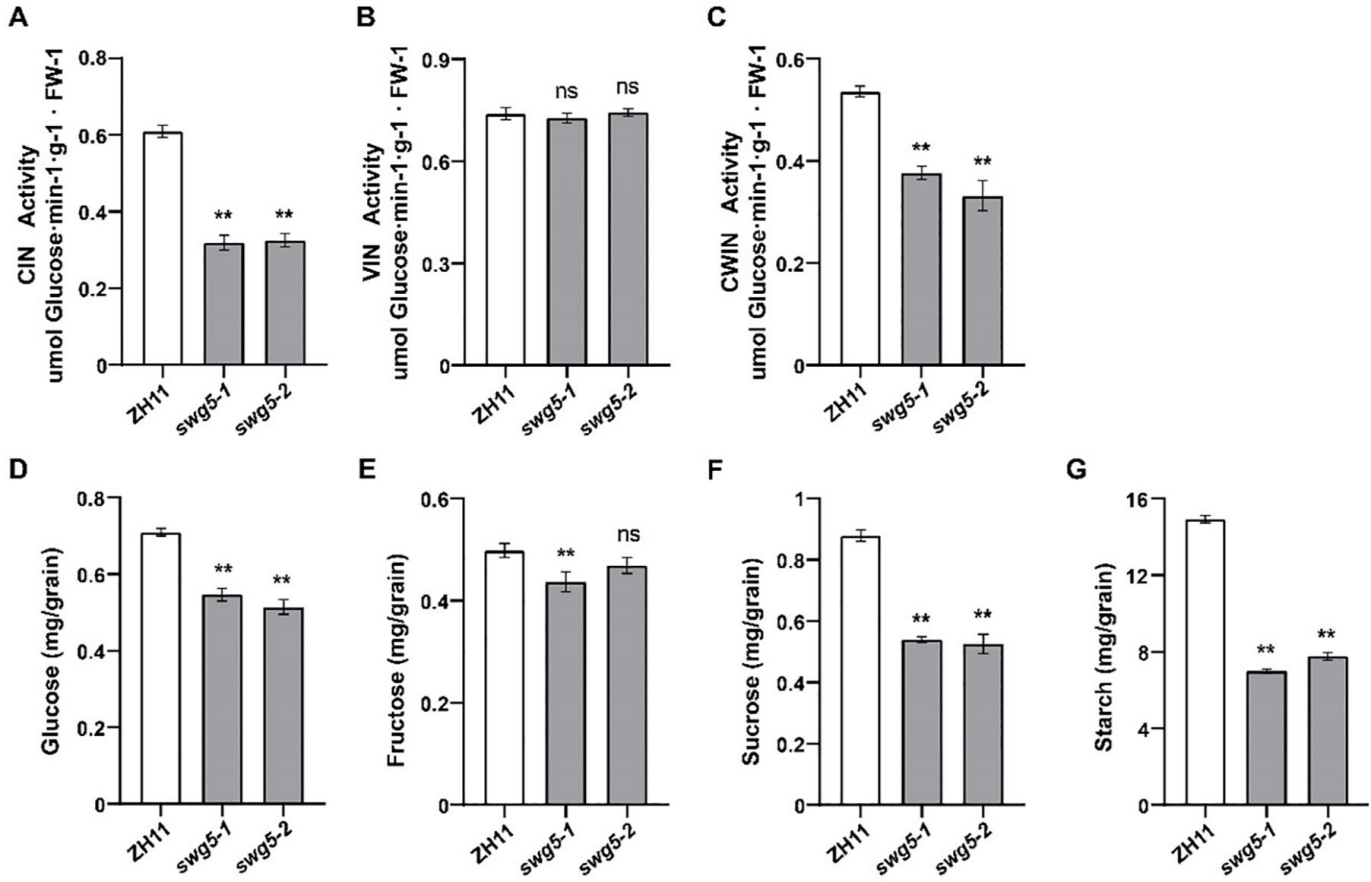
Comparison of enzyme activity and sugar and starch contents between ZH11, *swg5-1*, and *swg5-2*. **(A–C)** Activities of three invertase isoforms in young panicles of ZH11, *swg5-1*, and *swg5-2* plants. **(D–F)** Levels of glucose, fructose, and sucrose in young panicles of ZH11, *swg5-1*, and *swg5-2* plants. **(G)** Starch content in mature seeds of the ZH11, *swg5-1*, and *swg5-2* plants. Values are means ± SD. Student’s t-test was used to calculate the p-values; **P < 0.01. NS indicates no significant difference.

To determine if *SWG5* affects grain size and weight through sugar metabolism, we measured the activities of CIN, VIN, and CWIN, as well as sugar and starch content, in the young panicles of ZH11 and *SWG5* knockout mutants (*swg5-1* and *swg5-2*). The activities of CIN and CWIN were significantly lower in the *SWG5* knockout mutants than in ZH11 (**Figures 7A, C**). In contrast, the VIN activity showed no significant difference (**Figure 7B**). Sucrose, fructose, and glucose levels were also significantly lower in the knockout mutants, although fructose content in *swg5-2* was similar to ZH11 (**Figures 7D–F**). Additionally, the starch content in the mature seeds of *SWG5* knockout mutants (*swg5*-1 and *swg5*-2) was significantly reduced compared to ZH11 (**Figure 7G**). These results suggest that *SWG5* may regulate grain size by reducing invertase activity, which limits sucrose and starch accumulation.

## 4 Discussion

### 4.1 *SWG5* is a new allelic variant of *SRS3*

Rice grain size affects both yield and quality (Harberd, 2015; Li et al., 2018), and understanding such regulation is crucial for improving rice varieties and seed development (Yan et al., 2024; Zhang J. et al., 2023). Advances in molecular biology and genomics have accelerated research in this area (Long et al., 2024; Ren et al., 2023). However, the exact molecular mechanisms remain unclear.

In this study, we identified the *swg5* mutant from an EMS-mutagenized ZH11 population (**Figure 1**). Using MutMap, genetic complementation, and gene knockout techniques, we found that the candidate gene is *LOC_Os05g06280*, which encodes the kinesin-13a protein (**Figures 3**, **4**). *SWG5* is a new allelic mutant of genes such as *SRS3*, *SGL*, *BHS1*, and *sar1* (Deng et al., 2015; Kitagawa et al., 2010; Wu et al., 2014; Zhang et al., 2022). These mutants share traits such as small grains, short panicles, reduced plant height, and short internodes. The *SRS3* mutation occurs in the ninth exon of *LOC_Os05g06280*, with a single nucleotide change from C to T (Kitagawa et al., 2010). The *BSH1* mutation is in the eighth exon, with four nucleotide changes from CTGC to TTTT (Zhang et al., 2022). The *SGL* mutation is in the noncoding region upstream of the ATG start codon and involves a single base deletion (Wu et al., 2014). The *TCM2092* mutation occurs in the 11th intron, where a G to A change leads to abnormal splicing and truncates the *SWG5* protein from 819 to 762 amino acids (Kitagawa et al., 2010). In our study, we identified an *SWG5* mutation in the first intron, near the second exon, with a G to A change (**Figure 3B**). This mutation also causes abnormal splicing, leading to a truncated SWG5 protein of 819 amino acids. Additionally, the expression level of *SWG5* was reduced (**Figure 3E**). These mutations affect various phenotypic traits. We suggest that these changes may impact gene expression or function, affecting development, metabolism, and cellular processes. Further investigation is needed to fully understand the role of *SWG5* in rice.

### 4.2 *SWG5* has opposing effects on grain length and width

Many genes related to rice grain morphology have been identified (Li et al., 2019; Ren et al., 2023). Most of these genes affect either grain length (Wang et al., 2024, 2015) or width (Liu et al., 2017; Song et al., 2007) by regulating cell growth or expansion. However, most of these genes affect only one aspect of grain morphology. Some genes, such as *WTG1* and *GW6* (Huang et al., 2017; Shi et al., 2020), affect both traits. In this study, cytological analysis showed that the *SWG5* mutant had fewer longitudinal cells but no change in transverse cells (**Figures 2I, J**). This suggests that *SWG5* specifically regulates the direction of cell division, reducing the number of longitudinal cells and shortening the lemma length.

Additionally, cell size also plays a role in grain size. Despite having fewer cells, the *SWG5* mutant showed an increase in individual cell width, resulting in wider grains (**Figures 2B, H**). In conclusion, we propose that *SWG5* promotes cell proliferation to control grain length while limiting transverse cell expansion to control grain width. The underlying mechanism should be explored in future studies.

We used CRISPR/Cas9 gene editing to knock out *SWG5* in ZH11. The results showed that knocking out *SWG5* reduced grain length and 1000-grain weight but increased grain width (**Figure 4**). Previous studies have also indicated that *SWG5* plays a role in regulating salt stress tolerance (Chen et al., 2022). This result suggests that *SWG5* not only controls grain size but may also help plants grow and adapt to stress.

### 4.3 *SWG5* regulates grain size and weight through sugar metabolism

We performed an RNA-seq analysis on young panicles (5–10 cm) of ZH11 and *SWG5* knockout (KO) mutants to better understand how *SWG5* regulates grain size. KEGG analysis revealed that differentially expressed genes (DEGs) were enriched in pathways related to diterpenoid biosynthesis, amino sugar and nucleotide sugar metabolism, pentose and glucuronate interconversions, metabolic pathways, and the MAPK signaling pathway (**Figure 5B**). These results suggest that *SWG5* may regulate grain size through glucose metabolism.

Grain biomass accumulation depends on sucrose, which is converted into hexoses and other simple sugars as it moves from the leaf to the developing grain. These sugars are crucial for energy supply and assimilation (Koch, 2004; Wan et al., 2018). Sugar transporters are essential for carbon distribution, helping move sucrose from source leaves to growing tissues, while invertases break down sucrose (Li et al., 2017).

We further examined whether *SWG5* affected sugar metabolism by measuring enzyme activity and sugar composition in ZH11 and *swg5* mutants. Compared to ZH11, *swg5* mutants showed significantly reduced activity of CIN and CWIN in young panicles (**Figures 7A, C**). However, VIN activity did not differ (**Figure 7B**). Sugar composition in the KO mutants was also altered, with low levels of sucrose and hexoses (glucose and fructose) (**Figures 7D–F**).

Starch, the primary carbohydrate in rice grains, is synthesized from hexose units derived from sucrose and serves as the main energy storage in grains. We measured the starch content in mature grains of the *swg5* mutants, which was significantly lower (**Figure 7G**). This result suggests that the loss of *SWG5* reduces invertase activity, affecting sucrose breakdown and hindering grain biomass accumulation, ultimately leading to a smaller grain size. Other studies have also shown that multiple genes involved in sugar metabolism regulate grain size (Deng et al., 2020; Xu et al., 2019), further supporting the importance of sugars in grain development.

Although this study shows that *SWG5* plays a key role in regulating grain size, more research is needed. First, how *SWG5* controls grain growth through sugar metabolism, especially its interactions with sugar enzymes or transporters, requires further study. Additionally, the interaction between *SWG5* and other grain-size genes should be explored. Future work could involve creating double mutants of *SWG5* and other grain-size genes to examine their combined effects and to better understand the mechanisms of grain-size regulation.

## Data Availability

The original contributions presented in the study are publicly available. This data can be found here: NCBI, PRJCA034576.
