# Supplementary material for: SWG5 regulates grain size and weight via sugar metabolism-mediated signaling in rice
Source: Front Plant Sci. 2025 Mar 24;16:1552268. doi: 10.3389/fpls.2025.1552268 (PMC11977390; doi:10.3389/fpls.2025.1552268)
Supplement: Supplementary file 1 [file DataSheet1.pdf]

Table S1 Primers used in this study

Primer name	Primer sequence (5'-3')
Os04g0344100	F:CATTTTGAGTCTGTATGCGTGA
	R:TGCTGCTTTTCTGTTTACTTCC
Os03g0194900	F:TAACATCCATGGCATCCTGAAC
	R:GTGATGTGGAGGTAGAACCATG
Os05g0247100	F:CACAAGGATCTCTACTACGACG
	R:GTACCTGTTCCAGACCATGATG
Os02g0219200	F:CTGTCCCAAAGATGGTACCAT
	R:AATTCGTTCATGTGGTTCTCGT
Os03g0250400	F:AATCATCACTCGCGTTTTAACC
	R:GTGTAGTTCGCGAGATCTGATA
Os10g0159300	F:TGTTGATATGTTTGCGTGAAGG
	R:GACATGTAGGGATAGGCTTGAA
Os06g0696400	F:AGATCGACACGAGAAGATGATC
	R:TCAAACTTGGTACGTACATCGA
Os07g0539300	F:GATGAAGCCCATGATTGACTTC
	R:AAATCGTTGAATGCATAGGTCG
Os06g0356800	F:CATGGTCATCATGTCCTTCCTC
	R:GATGAACTGGCAGTGCTTGAT
Os04g0121100	F:TCACCTTGGGCGATAATCATAA
	R:CGCTCGAAAACTTGTATCTCAC
SWG5-RT	F:AGCTTACAGAAGTTCTTCGTGA
	R:CTTTTCTTGTATTGCTGCCCTT
SWG5-cDNA	F:ATGGGGGACTCCGGGGACGC
	R:GACAATGATAGGCAGACGAG
